# Hemodynamic determinants of left atrial strain in patients with hypertrophic cardiomyopathy: A combined echocardiography and CMR study

**DOI:** 10.1371/journal.pone.0245934

**Published:** 2021-02-10

**Authors:** Bhupendar Tayal, Maan Malahfji, John M. Buergler, Dipan J. Shah, Sherif F. Nagueh

**Affiliations:** 1 Methodist DeBakey Heart and Vascular Center, Houston, Texas, United States of America; 2 Department of Cardiology, Aalborg University Hospital, Aalborg, Denmark; University of Dundee, UNITED KINGDOM

## Abstract

**Background:**

Left atrial (LA) strain is associated with symptomatic status and atrial fibrillation in patients with hypertrophic cardiomyopathy (HCM). However, hemodynamic determinants of LA reservoir (LARS), conduit, and pump strains have not been examined and data are needed on the relation of LA strain with exercise tolerance in HCM.

**Methods:**

Fifty HCM patients with echocardiographic and CMR imaging within 30 days were included. Left ventricular (LV) volumes, mass, EF, scar extent, extracellular volume fraction (ECV), and LA maximum volume were measured by CMR. Echo studies were analyzed for mitral inflow, pulmonary vein flow, mitral annulus tissue Doppler velocities, LV global longitudinal strain, and LA strain. Twenty six patients able and willing to exercise underwent cardiopulmonary stress testing for peak oxygen consumption (MVO2), and V_E_/V_CO2_ slope. Patients were followed for clinical events.

**Findings:**

LARS was significantly associated with indices of LA systolic function, LV GLS, and LV filling pressures (P<0.05). Conduit strain was significantly associated with mitral annulus early diastolic velocity and ECV, whereas LA pump strain was determined by LA systolic function and indices of LV end diastolic pressure (all P<0.05). LARS and conduit strain were significantly higher in patients who achieved ≥80% of MVO_2_. LARS, conduit, and pump strains were significantly associated with atrial fibrillation (P<0.05).

**Conclusions:**

LV structure, systolic and diastolic function, and LA systolic function determine the 3 components of LA strain. LA strain is associated with exercise tolerance and clinical events in patients with HCM.

## Introduction

Hypertrophic cardiomyopathy (HCM) is the most common inherited cardiomyopathy and an important cause of sudden cardiac death and heart failure [1]. The presence of restrictive left ventricular (LV) filling is a risk factor for heart failure hospitalizations in HCM patients with normal LV ejection fraction (EF). Restrictive LV filling is characterized in part by reduced left atrial (LA) contribution to LV filling [2]. Further, LA size is a risk factor for death after surgical myectomy [3] and has been included in the risk model of sudden cardiac death by ESC [4]. Accordingly, there is ongoing interest in the assessment of not only LA size, but also LA function in patients with HCM. There are several indices of LA function that have been evaluated over the years including the changes in LA volume over the different phases of the cardiac cycle. Such indices were successfully explored to gain insight into LA function after alcohol septal ablation [5]. More recently, LA strain was examined and has been associated with symptomatic status [6]. However, the hemodynamic determinants of LA strain in patients with HCM were not evaluated, and there is a paucity of data with respect to LA strain association with LV structure and a given patient functional status. Accordingly, we sought to gain insight into the hemodynamic determinants of LA strain in patients with HCM, and their association with exercise tolerance and clinical outcomes.

## Methods

Consecutive HCM patients who underwent echocardiographic and CMR imaging within 30 days over the past 5 years and who met the inclusion criteria (below) were included in the study. Some of these patients were included in a previous report [7]. Inclusion criteria included an ejection fraction (EF) > 50%, sinus rhythm, no previous septal reduction therapy, and imaging by CMR and transthoracic echocardiography. The median difference in time between the 2 imaging studies was 3.5 days (0–21 days). Patients were excluded if they had coronary artery disease, the presence of pacemaker or defibrillator not compatible with CMR imaging, and more than mild aortic valve stenosis or regurgitation. There were no patients with mitral stenosis or moderate or severe mitral annular calcification. Heart rate and blood pressure were similar at the time of the two examinations. Imaging was performed after cardiac medications were withheld. The protocol was approved by Institutional Review Board of Houston Methodist Research Institute, and patients provided written informed consent.

Clinical evaluation included the assessment of NYHA functional class, the presence of angina and its severity, and the occurrence of syncope. Patients underwent symptom limited exercise treadmill testing on a modified Bruce protocol along with the measurement of peak oxygen consumption (MVO2), anaerobic threshold, RER (respiratory exchange ratio), and V_E_/V_CO2_ slope.

### Echocardiographic imaging and analysis

Patients were imaged with ultrasound systems equipped with a multifrequency transducer. Parasternal and apical views were acquired. Depth, gain, and transducer position were optimized to allow recording of complete LA volumes without foreshortening. In the apical four-chamber view, pulsed wave Doppler sample volume was placed at mitral valve annulus and tips to record mitral inflow at both locations. With color Doppler guidance, LV outflow tract (OT) gradient was recorded with continuous-wave (CW) Doppler. Pulsed wave tissue Doppler was applied to record mitral annulus velocities at septal and lateral sides of the mitral annulus. Peak velocity of tricuspid regurgitation (TR) was recorded by CW Doppler from multiple windows. Pulmonary regurgitation velocity, when available, was recorded by CW Doppler.

All measurements were performed by an observer blinded to clinical and CMR data. Mitral annulus diastolic diameter was measured in the apical views, and mitral annulus area was then calculated [8]. Peak LVOT gradient was derived with the modified Bernoulli equation as: LVOT gradient = 4*v*^2^, where *v* = peak velocity in LVOT by continuous-wave Doppler in m/s [9]. Mitral inflow was analyzed for peak early (E), late (A) diastolic velocities, E/A ratio, and deceleration time (DT) of mitral E velocity. LA ejection force was calculated using the equation: 0.5 × 1.06 × mitral annulus area × (peak A velocity)^2^ in kdyne [10]. In calculation of LA ejection force, absolute A velocity (peak A–E velocity at onset of A) was used when A velocity did not start at baseline. Pulmonary artery (PA) systolic pressure was derived as: (peak TR velocity by CW Doppler in m/s)^2^ + right atrial pressure [11]. Pulmonary artery (PA) diastolic pressure was derived as: (end diastolic PR velocity by CW Doppler in m/s)^2^ + right atrial pressure [11]. Right atrial pressure was estimated based on inferior vena cava maximum diameter and its change with respiration and hepatic venous flow [11].

Pulmonary vein flow was analyzed for peak velocity, duration, and velocity time integral of systolic (S), diastolic (D), and atrial reversal (Ar) velocities. The time difference between duration of pulmonary vein Ar velocity and mitral A velocity was computed (Ar-A) as an index of LV end diastolic pressure. A wave transit time, was measured between onset of A velocity at mitral inflow and onset of A velocity at LVOT with the sample volume located midway between LVOT and mitral valve. This time interval is shorter in the presence of increased LV stiffness and end diastolic pressure [12]. Mitral annulus early diastolic velocity (e’) was measured at septal and lateral sides of the mitral annulus (septal and lateral), and average E/e’ ratio was computed [12].

LV global longitudinal strain (GLS), and LA strain were analyzed offline using VVI (Syngo, Siemens) as the average of 3 beats using R-R gating. Mean interobserver difference for LA reservoir strain was 1.8±0.6%, whereas it was 2±0.8% for LA pump strain. All analyses were performed by a single observer blinded to clinical data, CMR and other echocardiographic measurements. Peak LA reservoir strain (LARS) during LV systole and peak LA pump strain were measured. LA conduit strain was then derived [13, 14]. LA stiffness index was obtained as the ratio of average E/e’ to LARS [12]. Statistical analysis was performed using LA strain values from the apical 4-chamber view [14].

### CMR imaging and analysis

CMR studies were performed on 3 Tesla MR system (Verio, Siemens Healthcare, Germany). Steady state free precession sequences were used to acquire a stack of short axis images at 1 cm interval during single breath holds, slice thickness of 6 mm with 4 mm gap, echo time = 1.33 ms, temporal resolution = 37.56 ms, flip angle 65° to 85°, repetition time 3.0 ms, phases = 20, field of view = 400 mm, field of phase = 81.3 m. Quantification of left and right ventricular ejection fraction (EF), volumes (end diastolic: EDV, end systolic: ESV and stroke volume: SV), and mass were calculated as per recommended guidelines [15]. The endocardial borders were manually traced for end diastolic and end systolic images for all slices. Papillary muscles were excluded from ventricular volumes. LA volume was determined using biplane area-length method [16].

Late gadolinium enhancement (LGE) imaging was performed 10–15 min after intravenous injection of a gadolinium contrast agent (gadopentetate dimeglumine or gadoterate meglumine, 0.15 mmol/kg). An EKG triggered inversion recovery gradient echo sequence was applied during a breath hold in long axis (4, 3, 2 chamber view slices) and short axis slices copying the scanning parameters of the corresponding cine images. The presence and extent of scar using LGE was performed offline by an experienced level 3 reader. Quantification of LGE was done according to the full width half maximum method which has excellent reproducibility [17].

The ECG triggered modified Look Locker inversion recovery (MOLLI) sequence was employed for assessment of myocardial T1 relaxation time in a mid-ventricular slice. Native myocardial T1 relaxation time was measured before administration of contrast. Acquisition scans for post contrast T1 mapping were performed after the standard delayed enhancement acquisition protocol for a mid-ventricular matching slice, approximately 15–20 min after the infusion of the contrast agent. Parameters for MOLLI technique include slice thickness of 6 mm, voxel size 2.1x1.6 x 6 mm, TE = 1.09, TR = 675, flip angle = 35^0^, 2-fold parallel imaging. Pre-contrast 5(4)2 and post-contrast 4(1)2(2)2 heartbeat sampling schemes were used on the 3.0-Tesla scanner. Shimming and delta frequency adjustments were applied to minimize off-resonance artifacts.

Extra cellular volume (ECV) fraction was assessed at 6 myocardial segments using post processing software cvi^42^ software, Circle cardiovascular imaging, Calgary, Canada. ECV fraction was calculated from the pre and post contrast images [18]. Briefly, an offline post processing analysis of T1 values was performed using manually contoured region of interests by a level 3 reader independent from the LGE analysis, for both pre and post contrast midventricular slices and ECV was calculated as: ECV = Δ R1 myocardium / Δ R1 blood × (100 − HCT), where Δ R1 myocardium or change in relaxation rate is given by: 1 / T1 myocardium post contrast injection– 1 / T1 myocardium pre-contrast injection, and Δ R1 blood = 1 / T1 blood-post contrast– 1 / T1 blood-pre contrast administration. By this technique an estimate of T1 is encoded in the intensity of each pixel. Using 17 segment model proposed by AHA, ECV_,_ T1 pre- contrast and T1 post contrast values were calculated separately for each of the six segments in the midventricular slice before and after contrast administration. Myocardial segments with any artifacts were excluded. For computing purposes, an average of global ECV was calculated weighted by the number of segments where it was possible to assess ECV. An offset of 20% from epicardial and endocardial contours was applied to the T1 sequences to reduce bias from partial volume effects.

### Outcome events

The study cohort was followed for the clinical events of atrial fibrillation, stroke, heart failure hospitalizations, and death. Mean follow-up was 77 months (6.4 years). Atrial fibrillation diagnosis was ascertained based on clinical records and EKG evidence of the arrhythmia. EKG evidence was based on recordings obtained during clinic visits, loop recorders, and Holter monitors.

### Statistical analysis

Numerical data are presented as mean ± standard deviation and categorical data as absolute number along with their percentage. Distribution of numerical data was tested for normality using Kolmogorov Smirnov test. Independent student t-test or Mann-Whitney U test were used to compare the numerical data, while chi-square test was applied to compare categorical data. A comparison between patients with and without significant LVOT obstruction at rest (LVOT gradient ≥ 30 mmHg) was performed for the complete dataset. Regression analysis was used to assess the correlates of LA strain with CMR and echo indices of LV and LA structure and function. Furthermore, these correlations were derived again adjusting for age and resting LVOT gradient using partial correlation. Univariable binomial logistic regression was performed for the study outcome using clinical and imaging parameters, including LA strain. LA strain was compared between patients who achieved ≥80% of predicted MVO_2_ for age and sex, and those who did not. Linear correlation was performed for the association of LA strain with V_E_/V_CO2_ slope. Significance was defined by two-tailed P <0.05. All statistical analyses were performed using R (version 3.5.0) and SPSS version 26 (IBM Corp., Armonk, N.Y., USA).

## Results

Table 1 presents a summary of clinical and exercise data of the study group. Most patients were symptomatic and were on beta-blockers and/or calcium channel blockers. The majority had abnormal LV and LA functions as reflected in LV GLS and LA strain measurements.

**Table 1:** Baseline clinical, exercise data, and LA strain of the study sample.

Variable	Total (n = 50)
Age, years	54.8±14.1
Gender (Men), n (%)	30 (60.0)
Hypertension, n (%)	29 (58.0)
Diabetes, n (%)	5(10)
Beta blocker therapy, n (%)	31 (62)
Calcium channel blocker therapy, n (%)	5 (10)
NYHA class, n (%)	
Class I	21 (42%)
Class II	15 (30%)
Class III-IV	14 (28%)
Angina n (%)	10 (20%)
ESC Score	2.5±1.6
**Exercise Data (N = 26)**	
Duration (min)	11± 5.1
Pre-stress HR, beats/min	64±13
Post-stress HR, beats/min	133±28
Pre-stress SBP, mm Hg	133±14
Pre-stress DBP, mm Hg	69±12
Post-stress SBP, mm Hg	162±19
Post-stress DBP, mm Hg	80±8
Anerobic threshold ml/kg/min	12.7±4.2
MVO_2_ ml/kg per min	21.2±7.4
Percentage of target MVO_2_ Achieved	79.8±25.9
MVO_2_ 80% of predicted achieved, n (%)	13 (50%)
Respiratory exchange ratio	1.1±0.1
V_E_/V_CO2_ slope	30.1±6.0
METs	6.7±3.9
**Strain Parameters**	
LV GLS (absolute %)	15.1±4.3
LA reservoir strain, %	35.3±17.1
LA conduit strain, %	23.6±13.0
LA pump strain, %	11.7±7.4

NYHA = New York Hear Association class, HR = heart rate, SBP = systolic blood pressure, DBP = diastolic blood pressure, MVO_2_ = peak myocardial oxygen consumption, V_E_ = minute ventilation, V_CO2_ = carbon dioxide production, LV = left ventricle, GLS = global longitudinal strain, LA = left atrium, ESC = European Society of Cardiology

### LV and LA structure and function

**Fig 1:**
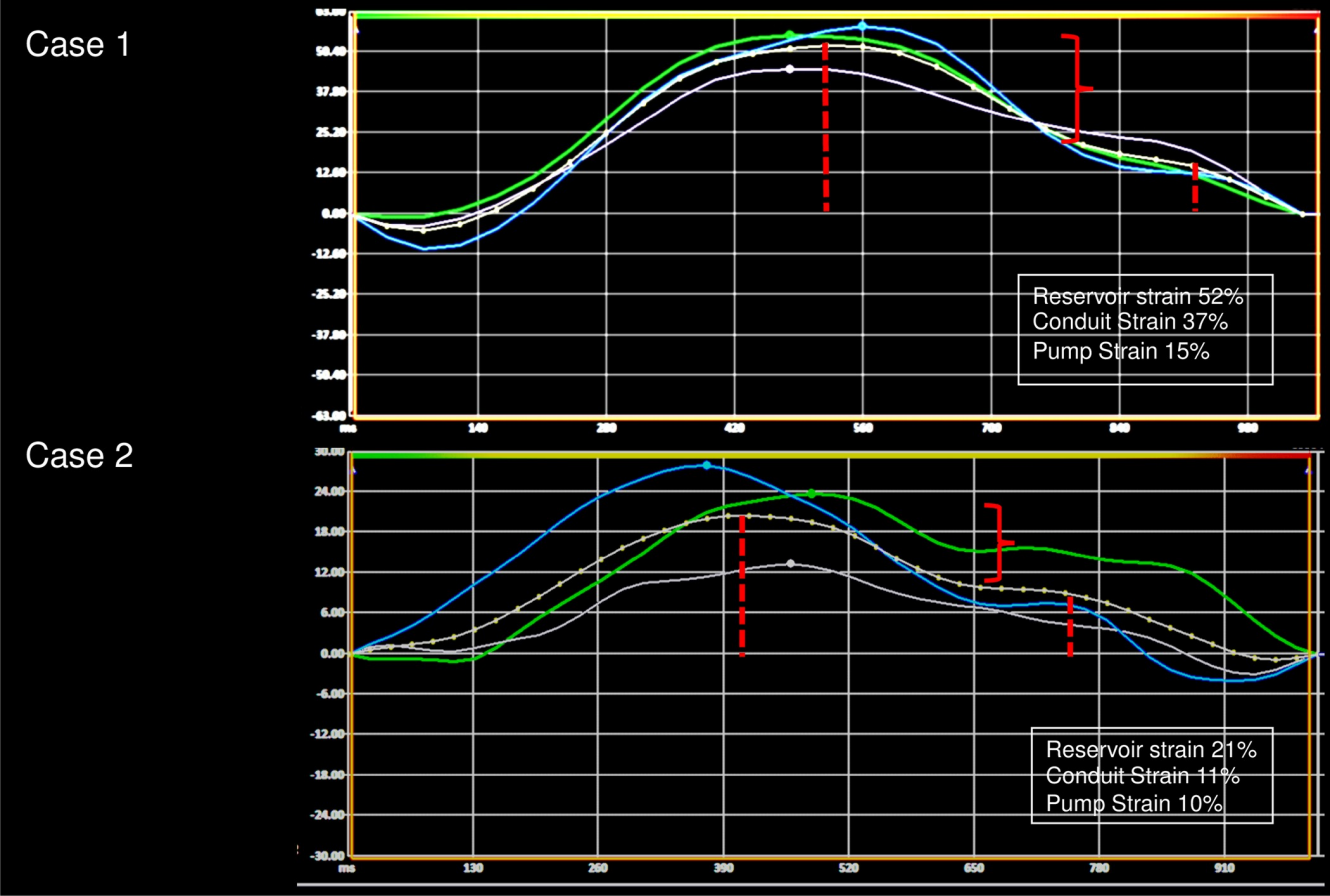
Examples of left atrial (LA) strain from a patient who achieved ≥80% of MVO_2_ (upper panel), and another patient who did not reach 80% MVO_2_ (lower panel).

A summary of LV volumes, EF, and mass is shown in Table 2. RV volumes and EF were normal. Most patients had asymmetric septal hypertrophy. LA maximum volume index by CMR was increased but there was no evidence of delayed hyperenhancement in the LA. LA maximum volume index was significantly larger in patients with dynamic obstruction (64±12 vs. 53±17 ml/m^2^, P = 0.01). Expectedly, myocardial scar was present in non-CAD pattern, and mean ECV fraction was 30%. Mitral regurgitation was present in 29 patients with an average regurgitant fraction of 30%. None of the patients had intrinsic mitral valve disease. There was a trend towards lower LV GLS and LA strain values (reservoir and conduit) in patients with dynamic obstruction compared to patients without, but the difference was not statistically significant. Examples of LA strain are shown in Fig 1.

**Table 2:** CMR imaging findings of the study sample.

Variable	Total (n = 50)	Variable	Total (n = 50)
LV end-diastolic volume (mL)	135.8±37.9		
LV end-diastolic volume index (mL/m^2^)	67.3±15.6		
LV end-systolic volume (mL)	33.7±18.5		
LV end-systolic volume index (mL/m^2^)	16.5±7.9		
LV cardiac output (Liters/min)	6.6±1.8		
LV mass (gm)	229.5±86.3	MR volume (mL)	32.4±20
LV mass index (gm/m^2^)	113.5±38.3	MR fraction (%)	29.8±11.8
LV Mass/Volume ratio (gm/mL)	1.7±0.6	LA diameter (cm)	4.4±0.8
LV stroke volume (mL)	102±24.2	LA volume (mL)	117.9±35.8
LV ejection fraction (%)	76.2±7.2	LA volume index (mL/m^2^)	58.6±16.0
RV end-diastolic volume index (mL/m^2^)	63.3±16.0	Septal thickness (cm)	1.9±0.6
RV end-systolic volume index (mL/m^2^)	23.6±9.5	Inferolateral wall thickness (cm)	1.1±0.3
RV ejection fraction (%)	63.5±8.6	Extracellular volume fraction (%)	29±3
LV hypertrophy pattern		LGE, n (%)	42 (84.0)
Apical Hypertrophy	4 (8.0)	LGE percentage	5.6± 5.7
Asymmetric Hypertrophy	44 (88.0)	Scar mass (gm)	14.5±18.2
Concentric Hypertrophy	2 (4.0)		

LV = left ventricle, MR = mitral regurgitation, LA = left atrium, LGE = late gadolinium enhancement, RV = right ventricle

### Hemodynamic determinants of LA Reservoir Strain (LARS)

**Fig 2:**
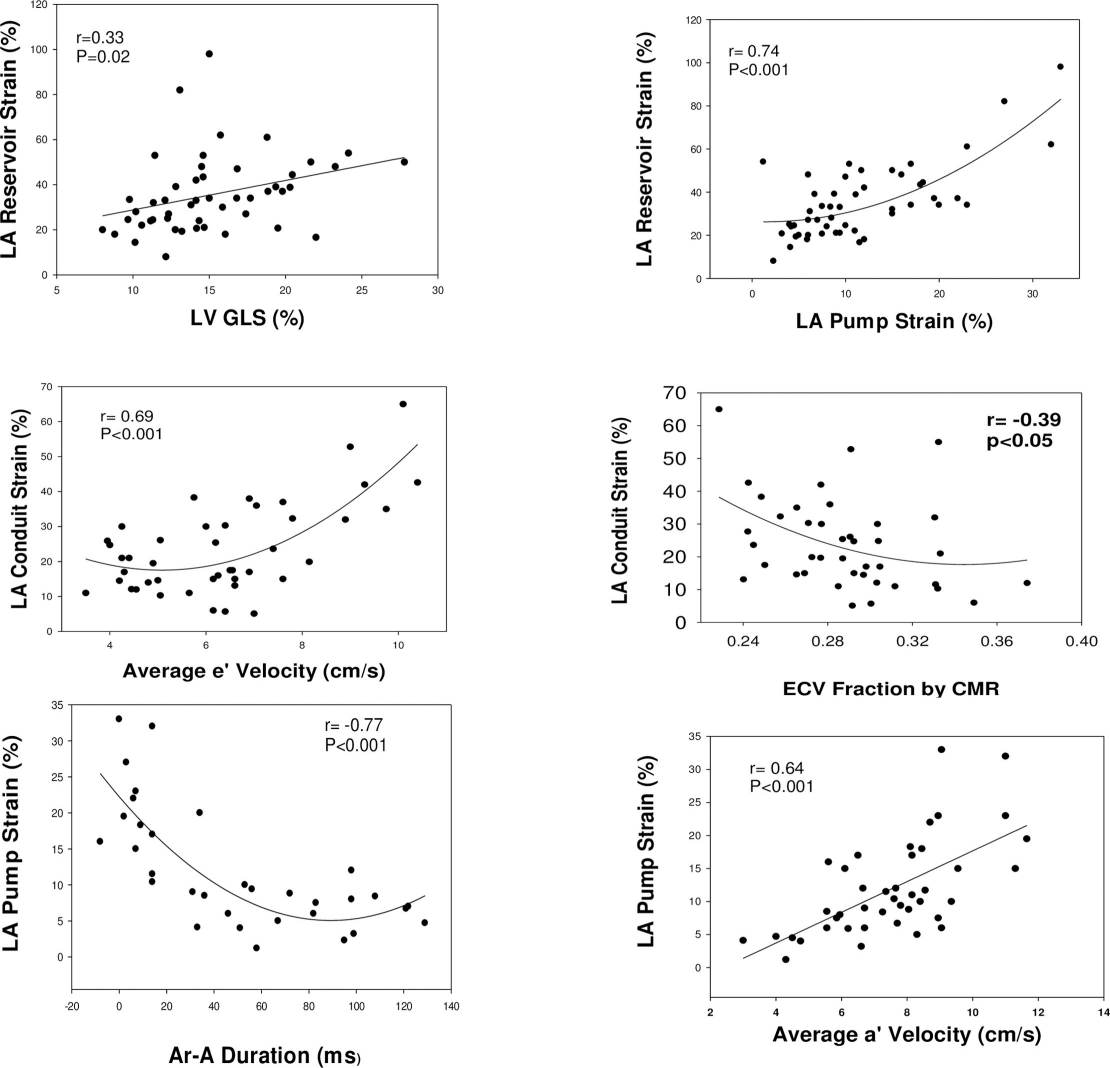
Upper row: Relation of LA reservoir strain to left ventricular global longitudinal strain (GLS) shown in the left panel and to LA pump strain in right panel. Middle row: Relation of LA conduit strain to mitral annulus early diastolic velocity (e’) shown in the left panel and to extracellular volume fraction (ECV) by CMR in right panel. Lower row: Relation of LA pump strain to the difference between pulmonary vein atrial velocity (Ar) duration and mitral inflow late diastolic velocity (A) duration shown in left panel. Relation of LA pump strain to average mitral annuls late diastolic velocity (average of septal and lateral velocities, (a’) by tissue Doppler shown in right panel.

LARS was related to LV GLS, such that patients with more LV systolic deformation had larger LARS values (Table 3). However, LARS was not related to LV volumes, mass/volume ratio, scar burden, or ECV. The strongest correlation was present between LARS and pump strain (r = 0.7, P<0.01, Fig 2). Likewise, significant direct relation was present between LARS and average a’ velocity by tissue Doppler. Indicators of elevated LV EDP had significant correlations with LARS (short A wave transit time is associated with increased LVEDP hence its direct relation with LARS). Mitral annulus e’ velocity had a significant direct relation. Reduced LARS was associated with elevated PA pressures, though the correlation did not reach statistical significance (Table 3).

**Table 3:** Correlations of left atrial strain parameters.

Variables	Number	Reservoir	Conduit	Pump
Left atrial volume	50	0.01	0.18	-0.3*
LV EDV	50	0.05	0.27	-0.35*
LV Mass	50	-0.02	0.06	-0.15
LV volume/mass	50	-0.1	-0.16	0.05
LV stroke volume	50	0.08	0.3*	-0.36*
E/E’ average	44	-0.24	-0.19	-0.21
E’ average	44	0.52***	0.69***	0.13
A’ average	43	0.4**	0.15	0.64***
IVRT	39	-0.16	-0.26	0.06
A wave transit time	48	0.39*	0.1	0.67***
Pulmonary vein A reversal velocity	33	-0.28	-0.22	-0.25
Ar-A duration	34	-0.51**	-0.36*	-0.77**
PA systolic pressure	19	-0.28	-0.24	-0.28
PA diastolic pressure	12	-0.42	-0.14	-0.51
LA ejection force	49	0.14	-0.01	0.49***
LV GLS	49	0.33*	0.3*	0.32*
ECV	40	-0.29	-0.39*	-0.03
scar mass	50	-0.04	-0.01	-0.07

LV = left ventricle, EDV = end-diastolic volume, IVRT = isovolumic relaxation time, PA = pulmonary artery, LA = left atrium, GLS = global longitudinal strain, ECV = extracellular volume fraction.

*<0.05

**<0.01

***<0.001

### Hemodynamic determinants of LA conduit strain

LA conduit strain was significantly and directly related to e’ velocity (Fig 2). LA conduit strain was higher in patients without scar than in those with scar, but the difference was not statistically significant (29±6.4% vs 22.6±11.8%, P = 0.19). On the other hand, LA conduit strain had a significant inverse relation with ECV, and Ar-A duration.

### Hemodynamic determinants of LA pump strain

LA pump strain was significantly and directly related to indices of LA systolic function including mitral inflow A velocity, mitral annulus a’ velocity, and LA ejection force. On the other hand, it was inversely related to (Ar-A) duration (Fig 2), and LV end diastolic volume. Similar to LARS, A wave transit time was directly related to LA pump strain. A wave transit time shortens with increased LV EDP, and thus the direct relation between A wave transit time and LA pump strain.

### Relation between LA strain and exercise tolerance

**Fig 3:**
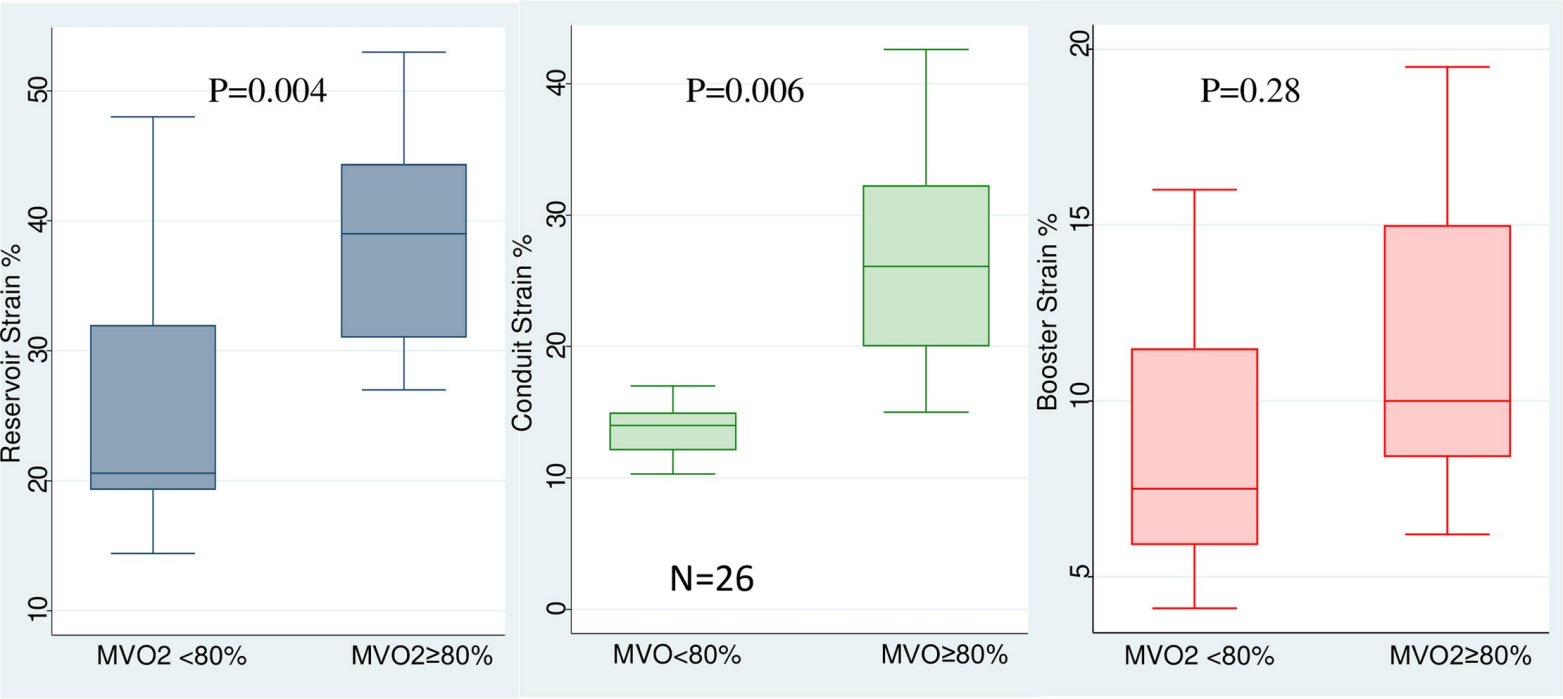
Box plots of LA reservoir (left), conduit (middle), and pump (right) strain of HCM patients who achieved ≥80% of MVO_2_, and those who did not reach 80% MVO_2_.

There were 26 patients who were able to and agreed to exercise. LARS and LA conduit strain, but not pump strain, were significantly higher in patients who achieved ≥80% of predicted maximum oxygen consumption than in those who did not (Fig 3). LA stiffness index was significantly lower in patients who achieved ≥80% MVO_2_, compared to patients who did not (0.16±0.03 vs 0.27±0.08, P<0.001). Further, LARS (β coefficient: -0.23, P = 0.02) and LA pump strain (β coefficient: -0.29, P = 0.009), but not conduit strain, were significantly related to V_E_/V_CO2_ slope.

### Relation of LA strain to clinical events

There were 11 patients who developed atrial fibrillation and one patient died (24% of patients had events). The were no strokes, or hospitalizations for heart failure. Table 4 shows the association of all variables with the outcome events. LARS, LA conduit, and pump strains were all associated with clinical events. The results were unchanged when atrial fibrillation was the only outcome considered. Table 5 shows the accuracy of the cutoff values of LA strain measurements in relation to outcomes.

**Table 4:** Factors associated with clinical events on univariate analysis.

Variables	Odds ratio (95% CI)	P value
Age	1.01 [0.97;1.07]	0.450
Hypertension	1.02 [0.27;3.80]	0.979
LV end-diastolic volume index	0.99 [0.95;1.03]	0.604
LV Mass Index	0.99 [0.98;1.01]	0.515
LVEF	1.00 [0.92;1.10]	0.919
LGE	2.48 [0.27;22.54]	0.419
IVRT	0.99 [0.95;1.03]	0.581
E’ avg	1.07 [0.73;1.57]	0.732
E/E’ avg	0.99 [0.89;1.09]	0.816
GLS	0.91 [0.76;1.08]	0.267
LA Ejection Force	0.62 [0.13;2.99]	0.556
A wave transit time	0.98 [0.95;1.02]	0.445
Average a’ velocity	0.98 [0.96;1.01]	0.281
LVOT gradient	1.01 [1.00;1.03]	0.107
LA Reservoir strain	0.86 [0.78;0.96]	0.005
LA Conduit strain	0.89 [0.81;0.98]	0.019
LA Pump strain	0.80 [0.66;0.96]	0.016
LA Stiffness index	5.43 (0.94;31.55)	0.059

LV = left ventricle, EF = ejection fraction, LGE = late gadolinium enhancement, ECV = extra cellular volume, IVRT = isovolumic relaxation time, GLS = global longitudinal strain, LA = left atrium, LVOT = left ventricular outflow tract

**Table 5:** Cutoff values for LA strain for the clinical events of atrial fibrillation and death.

Variable	AUC	Threshold	Sensitivity	Specificity
Pump	0.80 (95% CI 0.66–0.90, P = 0.0001)	≤6%	67%	87%
Conduit	0.76 (95% CI 0.62–0.87, P = 0.0006)	≤21%	92%	55%
Reservoir	0.86 (95% CI 0.73–0.94, P<0.0001)	≤27%	92%	76%

## Discussion

In patients with HCM, LARS is related to LV GLS, LV filling pressures, and LV elastic recoil. LARS is also related to LA pump function. LA conduit strain is determined by LV early diastolic recoil as inferred by mitral annulus e’ velocity and ECV. LA pump strain is primarily determined by LA systolic function and LA afterload. Patients who achieved 80% of MVO_2_ had higher LARS and LA conduit strain and lower LA stiffness index. V_E_/V_CO2_ slope was significantly related to LARS and LA pump strain. All LA strain measurements were related to clinical events.

### LA reservoir strain

LA reservoir strain is a measure of LA expansion during systole taking into account the initial volume of the atrium at the onset of systole. LA filling occurs as LA pressure falls below pulmonary vein pressure allowing for forward flow into the LA. Thus, the lower the LA systolic pressure, the higher forward systolic flow into the LA. The first phase of forward systolic flow occurs during LA relaxation with the drop in LA pressure. LA systolic and diastolic functions are tightly coupled [19, 20]. In an animal study, LA a’ velocity was directly related not only to LA dP/dt, but also to the invasive index of LA relaxation: [(Pa-Px)/Pa]/(tx-ta), where Pa refers to the peak pressure of LA a wave, Px refers to the trough of LA x wave, and (tx-ta) is the time interval between the x trough and peak of a wave [20]. Therefore, when LA contractility is normal, LA relaxation is preserved leading to lower LA pressure in early systole, increased LA systolic filling and thus higher LA reservoir strain. This explains the direct relation between LA reservoir and indices of LA systolic function in this study. The second phase of forward systolic flow occurs with LV contraction and mitral annulus descent towards the apex, leading to another drop in LA pressure (x’ drop in LA pressure recording) and hence the significant direct association shown here in HCM patients, and in patients with mitral valve regurgitation between LV GLS and LA reservoir strain [21]. In this study, the statistical association between LA reservoir strain and LV GLS was adjusted by age and LVOT gradient. This is likely due to the decrease of LV GLS with age and dynamic gradient which increases LV systolic pressure and thus afterload. Increase in LV afterload leads to lowering of LV GLS as GLS is dependent on loading conditions. At the same time LARS decreases with age and with dynamic obstruction which is often accompanied by increased LA pressure and lower LARS. LA reservoir strain is also dependent on LA stiffness and given the positive association between LA stiffness and LA pressure, it is not surprising to see the negative association between LA reservoir strain and the surrogates of LV filling pressures.

### LA conduit strain

LA conduit strain is a measure of the change in LA volume during early diastole. LA volume decreases in early diastole with transmitral flow into the LV. This occurs secondary to a drop in LV pressure in early diastole with LV relaxation and early diastolic elastic recoil. The significant and direct relation of LA conduit strain with mitral annulus e’ velocity shows the dependence of this metric of LA function on LV early diastolic properties, given the known association between e’ velocity and LV relaxation and elastic recoil [20, 22]. MR is expected to directly relate to LA conduit strain but since these patients had mostly mild MR, such a relation was not observed given the narrow range of MR volumes as measured by CMR. ECV as measured by CMR had a significant inverse relation with LA conduit strain which may be explained by the detrimental effect of increased fibrosis on LV diastolic function.

### LA pump strain

LA pump strain is directly dependent on LA systolic function. This is supported by direct and significant association with all indices of LA systolic function evaluated in this study, including mitral annulus a’ velocity, and LA ejection force. Of note in an animal study, LA a’ velocity was directly related to LA dP/dt [20]. Another significant determinant of LA pump strain shown in this report is LA afterload. LV late diastolic pressures reflect LA afterload and have a negative association with LA a’ velocity [20]. Likewise, both indices of LV end diastolic pressure: Ar-A duration and A wave transit time were significantly related to LA pump strain in this study. Interestingly, the strength of the correlation of LA pump strain with indices of LA systolic function was rather similar to its association with LV end diastolic pressure. Given the direct correlation between LA strain and LA systolic function, it may be more useful than other indices of LA function in predicting atrial fibrillation in HCM, similar to other patient populations [23].

### LA strain, exercise tolerance, and clinical events

Given the importance of LA function in maintaining normal LA pressure and adequate LV filling, LA strain was significantly associated with peak oxygen consumption and with V_E_/V_CO2_ slope as lower LARS and pump strain were accompanied by increased wasted ventilation. The observation in patients with HCM is similar to that in other patient populations where LARS was associated with exercise tolerance [24]. Given the sample size, additional studies are needed to corroborate these findings which if true would lead to the recommendation of taking account of all LA strain measurements: reservoir, conduit, and pump in HCM patients.

Likewise, the association noted in this study of LA strain with clinical events, mostly atrial fibrillation, is similar to other studies showing the association of LA volumes by echocardiography with outcomes in patients with HCM [3, 25], and LA reservoir and conduit strain with paroxysmal atrial fibrillation [26, 27].

### Limitations

Most of the patients did not undergo cardiac catheterization and thus the evaluation was limited to noninvasive measurements of LV and LA hemodynamics. Notwithstanding, the noninvasive measurements obtained in this study have all been validated against the invasive standards, including invasive indices of LA systolic function and LA relaxation [12, 20], LV systolic function, and LV end diastolic pressure [28–30]. Only 50 patients were enrolled primarily due to the inclusion criterion of enrolling only patients with both CMR and echocardiographic imaging within 30 days. While we have shown associations, given the pathophysiological implications of these associations, we believe the study contributes to gaining insights into the hemodynamic determinants and the clinical relevance of LA strain. ECV was measured at midventricular level and could be different in apical segments in patients with apical hypertrophy. Therefore, we may have underestimated the extent of ECV in the four patients with apical HCM. There were few events in the study which limits the ability to address the incremental value of LA strain over clinical and other echocardiographic measurements. However, only LA strain was associated with clinical outcomes and the findings are very similar to other studies in HCM patients which included more patients and more events [26, 27]. Given the small number of events and the concerns with collinearity in multivariable models, multivariable analysis was not pursued, and we could not address which of the strain measurements has the most prognostic value.

### Clinical implications

LA reservoir strain has been advocated as an index of LA pressure based on clinical studies showing its significant association with mean wedge pressure [12, 13]. However, as noted in this study, LA reservoir strain is not a pure metric of LA pressure since it is significantly affected by LA systolic/diastolic properties and LV systolic function. Therefore, caution should be exercised when drawing inferences about LA pressure based on LA reservoir strain in HCM.

LA conduit strain is an interesting parameter that merits further evaluation as it provides another index of LV early diastolic recoil, albeit an indirect index. It remains to be seen how it relates to LV early diastolic properties in the presence of significant MR. LA pump strain is an index of LV end diastolic pressure [13], albeit also dependent on LA systolic function. Using multivariable regression models, it is possible to draw inferences about LV late diastolic pressures once LA systolic function is accounted for. Further, LA pump function may prove to be an important predictor of atrial fibrillation and stroke in HCM. These findings likely apply to most patients with cardiac disease but additional studies are needed for confirmation.

There are recent attempts aimed at standardizing LA strain analysis that when adopted by most labs can increase the feasibility and consistency of LA strain measurements [14]. Given the fact that LA strain can now be readily obtained and measured online on most ultrasound systems, it is possible to include its value in the standard report and to consider it in reaching recommendations pertaining to patient management, particularly LA reservoir and pump strain in HCM patients for prediction of atrial fibrillation with potential inclusion in future guidelines for risk stratification and management.

Further for LA strain to be useful clinically in a given patient, the value should be outside the normal range. A recent meta-analysis [31] in 2542 normal subjects reported the normal value for LA reservoir strain at 39% with 95% confidence intervals of 38–41%. The corresponding value for LA conduit strain was 23.6% (95% CI: 21–25%), and for LA pump strain was 17% (95% CI: 16–19%). Of note, the mean values for LARS (35.3%), and LA pump strain (11.7%) in this group of HCM patients were less than the lower 95% CI for the corresponding values in the normal population, while LA conduit strain mean value was similar to that in normal subjects.

## Supporting information

S1 Data (XLSX)
